# Reading Text Increases Binocular Disparity in Dyslexic Children

**DOI:** 10.1371/journal.pone.0027105

**Published:** 2011-11-04

**Authors:** Julie A. Kirkby, Hazel I. Blythe, Denis Drieghe, Simon P. Liversedge

**Affiliations:** 1 Psychology Department, Bournemouth University, Bournemouth, United Kingdom; 2 School of Psychology, University of Southampton, Southampton, United Kingdom; Macquarie University, Australia

## Abstract

Children with developmental dyslexia show reading impairment compared to their peers, despite being matched on IQ, socio-economic background, and educational opportunities. The neurological and cognitive basis of dyslexia remains a highly debated topic. Proponents of the magnocellular theory, which postulates abnormalities in the M-stream of the visual pathway cause developmental dyslexia, claim that children with dyslexia have deficient binocular coordination, and this is the underlying cause of developmental dyslexia. We measured binocular coordination during reading and a non-linguistic scanning task in three participant groups: adults, typically developing children, and children with dyslexia. A significant increase in fixation disparity was observed for dyslexic children solely when reading. Our study casts serious doubts on the claims of the magnocellular theory. The exclusivity of increased fixation disparity in dyslexics during reading might be a result of the allocation of inadequate attentional and/or cognitive resources to the reading process, or suboptimal linguistic processing per se.

## Introduction

Despite normal intelligence and educational opportunities, some children show a persistent difficulty in learning to read; these children are often diagnosed with developmental dyslexia [1]. The most widely accepted theory of dyslexia is the phonological deficit theory [2], [3], [4], in which it is argued that children with dyslexia have a cognitive-level deficit in representing phonological information. The theory stipulates that underspecified phonological representations and difficulties in associating printed letters with relevant speech sounds cause the reading difficulties experienced by these individuals.

There are some studies, however, that have shown abnormalities in the magnocellular pathway of the brain in dyslexic individuals [5], [6], [7], [8] and some researchers have suggested that visual impairment, rather than a linguistic processing problem, causes dyslexia [8], [9]. The magnocellular deficit theory purports that children with dyslexia have poor binocular coordination (stemming from the failure to develop a dominant eye), which causes problems in obtaining a single, stable perceptual representation of words from the two retinal inputs [9]. Erkelens [10], however, showed greater caution in assessing a link between binocular coordination and magnocellular function by stating “It is speculated that magnocellular layers process disparities that drive vergence and that a parvocellular stream of disparity processing is involved in depth perception”. Previous research has aimed to characterise the nature of inappropriate binocular eye movements associated with dyslexia [11]. In fact, there are often anecdotal reports that dyslexic readers experience blurring of letters, letters moving around in a word, and letters obscuring one another. Therefore, the possibility that atypical fixation disparity disrupts the visual percept of the fixated word for children with dyslexia fits well with these anecdotal reports. Some researchers have even claimed that patching one eye for a period of time helps to stabilize the child's ocular dominance, which consequently improves reading ability [12], [13], [14].

Three major criticisms can be leveled at the claims of the magnocellular theory. First, there is little direct evidence demonstrating that children with dyslexia experience “poor” binocular coordination [11]. There is a paucity of studies that have actually measured the positions of the two eyes in relation to each other; many have used subjective tasks and conclusions with respect to binocular coordination were inferred [15], [16].

Second, the magnocellular theory associates binocular alignment with reading ability, with the clear implication that perfect alignment is the “normal” end state for development of binocular control during reading [9]. Previous studies have demonstrated, however, that the two eyes typically fixate more than one character space apart on around half of all fixations during reading without causing reading difficulties in adults [17], and even larger disparities are observed in typically developing children up to the age of 10 to 11 years [11], [18]. Thus, the inference that perfect binocular alignment is necessary or normal for unimpaired reading conflicts with eye movement data from adults and typically developing children.

Third, any relationship that may exist between binocular coordination and dyslexia might not be causal, but may be a correlation or consequence of reading difficulties. Linguistic processing difficulties can, themselves, cause disruption to eye movement behavior [19], [20]. Several studies have demonstrated a disruption to typical monocular eye movement behavior for individuals with reading difficulties [21], and it is also possible that linguistic processing difficulty experienced by children with dyslexia might be reflected in measures of binocular coordination.

Recently, Jainta and Kapoula [22] provided interesting empirical data concerning the relationship between binocular coordination and developmental dyslexia. In their study, binocular eye movements were recorded from thirteen children with dyslexia while they read six eight line passages of text; these data were then compared to similar data from seven age-matched, control participants under identical experimental conditions. Jainta and Kapoula reported increased disconjugacy during saccades in dyslexic compared to non-dyslexic children, and showed that post-saccadic drift movements observed in dyslexic children were uncorrelated to the magnitude of fixation disparity. Furthermore, they reported larger standard deviation in fixation disparity for dyslexic children compared to non-dyslexic children. Jainta and Kapoula conclude that, “…besides impaired phonological processes – visual/ocular motor deficits exist in dyslexics which might perturb the fusional process”. While Jainta and Kapoula's data are extremely interesting, and their conclusions warranted, their study is silent in relation to the question of whether poor binocular coordination is causative in relation to poor reading performance observed in developmental dyslexia.

Following the magnocellular theory, if dyslexia is caused by general impairment of binocular coordination, then increased fixation disparity will be observed in dyslexic children compared to skilled readers (TD children and adults) during both reading and a nonlinguistic task that elicits eye movement behavior very closely approximating that which occurs during reading. Such effects would be consistent with visual impairment being crucial in the pathogenesis of developmental dyslexia [9]. Alternatively, and consistent with theoretical claims that dyslexia causes disruption to eye movement behavior during reading [21], increased fixation disparity should occur during reading, but not non-reading tasks. Thus, in the present study, we attempted to assess the extent to which poor binocular coordination plays a significant role in the etiology of developmental dyslexia by recording eye movements in three participant groups – skilled adult readers, typically developing children, and children with dyslexia during two experiments, a reading *and* a dot scanning task (eliciting reading-like saccades and fixations in the absence of linguistic processing).

## Results

For both experiments, data were analyzed by means of linear mixed effects modelling specifying participants as random factors. For the reading experiment the sentence being read was also entered as a random factor. For the dot scanning experiment the number of dots in the dot string was entered as a random factor. Standard procedures were employed in the construction of the initial models in that all factors potentially influencing binocular disparity as suggested by prior research were entered into the model: participant group, the amplitude of the incoming saccade and fixation position relative to the centre of the screen; (see Table 1 for the basic characteristics of eye movements during reading and dot scanning, obtained in the current experiments). Comparisons between the initial and reduced models were carried out to obtain the most parsimonious model that was not statistically inferior in terms of fit of the data relative to the initial model.

**Table 1:** Basic characteristics of eye movements during reading and dot scanning: mean fixation durations, saccade length, regression frequency, number of fixations and total reading time (standard deviations in parenthesis).

	Fixation duration (ms)	Saccade length (characters)	Regression frequency(%)	Number of fixations	Total reading time (ms)
Adults					
Reading	195 (81)	7.3 (5.1)	18.6 (8.8)	9 (2.8)	1741 (572)
Dot scanning	434 (231)	4.3 (3.5)	15.5 (3)	9 (4.6)	
TD children					
Reading	231 (104)	6.4 (4.9)	26.2 (5.8)	13 (4.3)	3105 (1091)
Dot scanning	378 (222)	4.4 (3.4)	13.5 (3)	11 (6.1)	
Children with dyslexia					
Reading	244 (123)	5.5 (5.0)	28.4 (7.1)	16 (6.1)	4075 (1695)
Dot scanning	374 (214)	4.3 (3.3)	15.5 (3)	11 (5.3)	

Note: The total reading time in the dot scanning experiment was determined by the pre-set trial duration.

### Experiment 1 (dot scanning)

Mean binocular disparity for adults was 0.24°, which was significantly different from 0° (*t* = 4.418, *p*<0.001; Table 2). There were no differences between adults and typically developing children (0.34°; *t* = 1.444, *p* = 0.148), or between adults and children with dyslexia (0.28°, *t*<1). Further, a contrast directly comparing the typically developing children and the children with dyslexia showed no significant differences in start of fixation disparity (t<1). Thus, children with dyslexia exhibited fixation disparity during the dot scanning experiment that was comparable to that of adults and typically developing children.

**Table 2:** Start of fixation disparity for all valid fixations during the dot scanning experiment: coefficients and standard errors are shown and the t-value with significance.

Predictor	Coefficient	Std. Error	t value
Intercept (Adults)	.239	.054	4.418***
TD children	.106	.074	1.444
Children with dyslexia	.037	.073	0.501
Position on screen	−.005	.004	−1.185
TD children X position screen	.033	.005	6.298***
Children with dyslexia position screen	.014	.005	2.670**

While we did not find a significant main effect of position on the screen relative to the centre there was a significant interaction found for child participants and the position of fixation on screen. Fixation disparity increased by approximately 0.03° relative to the position of fixation on the screen for child participants (*t* = 7.34, *p*<0.001); however this effect was not as pronounced for children with dyslexia (*t* = 3.09, *p*<0.01). This effect was very small, and was not the primary issue of interest in our experiment.

### Experiment 2 (reading)

**Figure 1:**
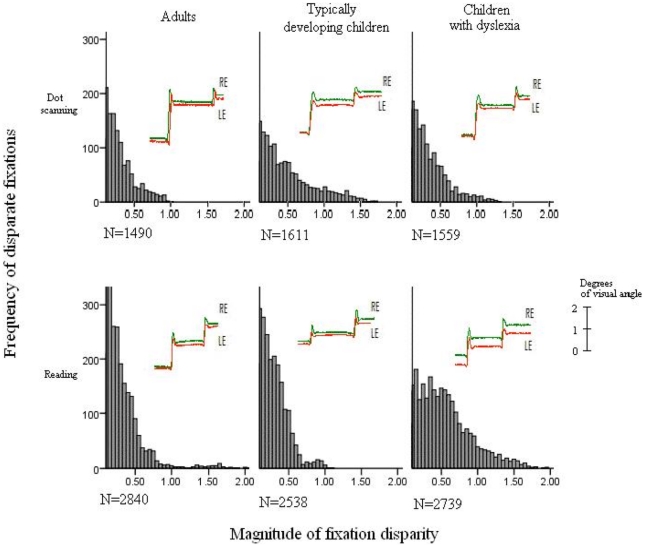
Frequency of disparate fixations; binocular eye movement traces representative of the mean fixation disparity for each group.

Mean binocular disparity for adults was 0.25° which was significantly different from 0° (*t* = 4.208, *p*<0.001, Table 3), and again there was no difference between adults and typically developing children's binocular disparity (0.22°, *t*<1). Critically, the mean binocular disparity in children with dyslexia was 0.22° larger (0.48°, *t* = 2.459, *p* = 0.013) than that observed for typically developing children. Additionally, a contrast directly comparing the typically developing children and the children with dyslexia showed that children with dyslexia exhibited an increase of 0.25° in start of fixation disparity compared to typically developing children (t = 2.49, p<.05). Thus, while adults and typically developing children exhibited similar magnitudes of fixation disparity in both experiments, children with dyslexia experienced significantly poorer binocular coordination (substantially increased disparity) when reading compared to adults and non-dyslexic children (see also Figure 1).

**Table 3:** Start of fixation disparity for all valid fixations during the reading experiment: coefficients and standard errors are shown and the t-value with significance level.

Predictor	Coefficient	Std. Error	t value
Intercept (Adults)	0.251	.060	4.208***
TD children	−0.030	.091	−0.325
Children with dyslexia	.224	.091	2.459*
Position on screen	−.003	.003	−1.080
Incoming saccade length	.010	.003	3.075**
TD children X position screen	.012	.004	2.880**
Children with dyslexia X position screen	.010	.004	2.517*

A small main effect of incoming saccade length on the magnitude of fixation disparity was found; this had an impact of 0.01° on the intercept and was consistent for all three participant groups. This pattern of results showed that the greater the amplitude of the incoming saccade, the greater the disparity on the subsequent fixation [23]. Note that while this effect was significant it was also quite small; this was not surprising as saccadic amplitudes are not typically very large during reading. Again there was no main effect of fixation position on screen, however, there was a significant interaction with participant group; the positive coefficient indicates that the magnitude of fixation disparity increased by approximately 0.01° for child participants when they fixated the stimuli presented at the far sides of the screen compared to when they fixated the stimuli presented in the centre of the screen (*t* = 2.39, *p*<0.05). This effect was not significantly different for the two groups of children (*t*<1).

### Analyses of a subgroup of children from the dyslexic group

The subset of children comprised of six children from the dyslexic group whom participated in both experiments. These children had a mean age of 10.14 years in Experiment 1, and 11.78 years in Experiment 2. On average, therefore, the children were a year older when they participated in the reading experiment. Note that the broad developmental trend is for fixation disparity to decrease with age [11], [18]. Within-participant analyses were conducted on this subset of data to directly examine the effect of reading on binocular coordination in children with dyslexia. Compared to dot scanning (mean = 0.25°), the task demands associated with reading increased binocular disparity by 0.30° (mean = 0.55°, *t* = 2.285, *p* = 0.02; Table 4), an increase of more than a full character space.

**Table 4:** Fixation disparity during reading and dot scanning experiments (for the subgroup of dyslexic children, n = 6, who participated in Experiments 1 & 2): coefficients and standard errors are shown, and t-values with significance levels.

Predictor	Coefficient	Std Error	t value
Intercept (dot scanning task)	.246	.149	1.654
Reading task	.302	.132	2.285*
Position on screen	.013	.003	3.776***

## Discussion

In the present research, we aimed to investigate whether poor binocular coordination might be causally related to the reading difficulties experienced by children with dyslexia. The results from the study showed: (*i*) children with dyslexia have an increased magnitude of fixation disparity when they are reading compared to dot scanning – within the same group of children, binocular coordination was affected by the task; (*ii*) in comparison with other participant groups, when reading, the magnitude of fixation disparity was significantly greater in the dyslexic children; and (*iii*) adults' and typically developing children's binocular coordination was equivalent, and this was the case during both reading and dot scanning. This pattern of results formally demonstrated that when children with dyslexia were required to read sentences, the magnitude of fixation disparity was greater than that found when they were scanning simple dot stimuli.

The literature concerning adult binocular eye movements is substantial, and the basic characteristics of the two eyes' coordination during reading are well documented [17], [18], [24]–[28]. These studies have shown that disparity between the points of fixation is commonplace during reading and the magnitude of disparity often extends more than one character space but rarely more than two. In the present study, complementary to the published literature, the adults' fixation disparity was shown to be significantly greater than 0°.

In contrast, both comparisons between-groups and within-group for the two tasks showed that children with dyslexia exhibited increased fixation disparity when reading compared to when dot scanning. The current results provide compelling evidence that children with dyslexia have a stimulus-specific deficit in regard to binocular coordination. Importantly, as increased binocular disparity was observed for dyslexic children exclusively during reading, this precludes the conclusion that dyslexia is caused by poor binocular coordination. Previous research has shown that contrast dependence of the horizontal vergence response is consistent with mediation by the parvocellular pathway [29] further weakening the suggestion that problems in binocular coordination cause dyslexia, via a disruption in the magnocellular visual pathway. In fact, our pattern of effects is consistent with the conclusion that the causal link may be in the opposite direction, where atypical binocular coordination is a consequence of the reading task. This raises the question of which aspects of the stimulus or task demands during reading cause fixation disparity to increase in children with dyslexia. There are two likely types of explanation.

**Figure 2:**
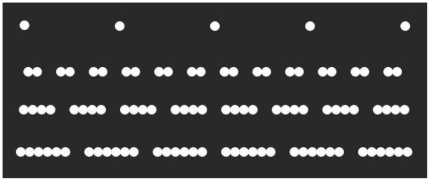
Stimuli used in Experiment 1. Panel shows a single dot trial; a two dot string trial; a four dot string trial; a six dot string trial; only one row of stimuli was presented in a trial.

**Figure 3:**
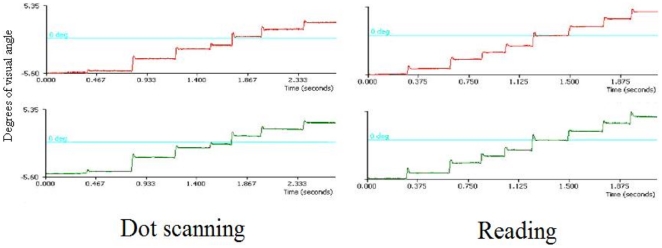
Raw eye movement data showing the typical “step-like” pattern of eye movements during the dot scanning and reading experiment. Horizontal axes represent trial duration in seconds. Vertical axes represent degrees of visual angle.

First, it is possible that the visual characteristics of the text are somehow related to increased fixation disparity for children with dyslexia. The gross visual characteristics of the sentence and dot stimuli were carefully designed in order to elicit comparable oculomotor behaviour (see Figures 2&3). There were, however, more fine-grained differences in the visual characteristics of the sentences compared to dot strings. Sentences (and their constituent words and letters) contain greater variability in spatial frequency, orientation, luminance, and target size than do rows of dot strings. Research with adults has shown that the characteristics of the visual stimulus affect fusional limits [30], [31].

Second, the two tasks differed in terms of the cognitive demands and linguistic processing required. Reading is a complex task which requires substantially more cognitive processing than scanning dot strings. Standard eye movement research has demonstrated that linguistic processing difficulty is reflected in monocular eye movement behaviour in children with dyslexia, for example: longer fixations, more fixations and shorter saccades [21]. Indeed, we observed such patterns in the data from the present reading experiment (Table 1). We, thus, consider it possible that processing difficulties experienced by children with dyslexia may also underlie the observed differences in binocular coordination between the reading and dot scanning tasks. A likely possibility is that increased cognitive demands for the dyslexic readers may have led, directly or indirectly, to reduced engagement of attention during the reading task. Further research is required, however, before any strong conclusions can be formed as to the influence of either the visual complexity of the stimulus and/or the cognitive demands of linguistic processing on binocular coordination in children with dyslexia.

In conclusion, the disruption to typical oculomotor control during reading observed in children with dyslexia in the present experiments is considered to reflect the individuals' difficulty with linguistically processing printed text; this oculomotor disruption may also include increased fixation disparity. Our results indicate that less precise binocular coordination in dyslexic readers may reflect issues associated with differences in the visual characteristics of written text compared to simpler stimuli, or increased cognitive demands (and potentially, attentional disengagement) during processing of linguistic compared with non-linguistic information. Importantly, however, poor binocular coordination is unlikely to play a causal role in these children's reading difficulties. Clearly, our data represent a stimulus specific-deficit in regard to binocular coordination.

## Methods

### Ethics Statement

The investigation adhered to the principles of the Declaration of Helsinki, and was approved by the Ethics Committee at the School of Psychology, University of Southampton for human experimentation. Informed oral consent was obtained from each child, in addition to the written consent obtained from parents, after explanation of the procedure of the experiment.

### Participants

In Experiment 1 there were eight adult participants with a mean age of 23.78 years (SD: 3.50), eight typically developing children with a mean age of 9.89 years (SD: 0.70) and eight dyslexic children with a mean age of 10.41 years (SD: 0 .84). There was no significant difference in the age of the two groups of children (*F* (1, 14) = 1.76, *p* = 0.21). In Experiment 2 there were eleven adult participants with a mean age of 21.09 years (SD: 3.05), eight typically developing children with a mean age of 10.67 years (SD: 1.1), and eight dyslexic children with a mean age of 11.38 years (SD: 1.1). Again there was no significant difference between the age of the two groups of children (*F* (1, 14) = 1.51, *p* = 0.24).

### Criteria for inclusion in dyslexic group

Prior to recruitment all dyslexic children had a formal, independent diagnosis of dyslexia, either through their Local Education Authority Psychology services or through Dyslexia Action. A standardised reading test was also conducted and results were consistent with their formal diagnoses; reading achievement was substantially below that predicted from their chronological age, while their IQ scores fell within the normal range on a standardised intelligence test (IQ≥90). The children with dyslexia had a mean discrepancy of 3.5 years between their chronological age and their measured reading age in Experiment 1 (*t* (7) = 13.79, *p*<0.001), and a mean discrepancy of 4.5 years in Experiment 2 (*t* (7) = 8.98, *p*<0.001). See Table 5 for formal comparisons of the typically developing and dyslexic child participants.

**Table 5:** Means (SD in parentheses) for the WASI IQ test, WIAT reading tests, and exception word reading: all t-tests were two-tailed.

Experiment 1. Dot scanning		
	Typically developing children	Children with dyslexia
IQ	118.25 (5.95)	108.63 (12.49), *p* = .07
Word reading	105 (9.51)	77.25 (11.88), *p*<.001
Comprehension	118.13 (6.55)	99.75 (13.48), *p* = .004
Pseudoword reading	107.62 (9.53)	83.62 (7.69), *p*<.001
Exception word reading	39.75 (3.95)	30.37 (5.42), *p* = .001

Experiment 2. Reading		
	Typically developing children	Children with dyslexia
IQ	112.12 (15.53)	106.00 (12.19), *p* = .395
Word reading	107.50 (7.95)	76.37 (11.22), *p*<.001
Comprehension	118.25 (9.80)	98.62 (12.98), *p* = .004
Pseudoword reading	109.00 (5.65)	80.62 (3.24), *p*<.001
Exception word reading	42.12 (2.59)	30.75 (6.25), *p*<.001

### Off-line reading ability and IQ measures

Reading and IQ were measured by means of the following off-line tests: (*i*) Two subtests of the Wechsler Abbreviated Scale of Intelligence [32]: a) the vocabulary subtest; b) the matrix reasoning subtest. (*ii*) The reading subtests of the Wechsler Individual Achievement Test [33]: a) word reading: b) pseudoword decoding; c) reading comprehension. (*iii*) Exception word reading, where they were asked to read aloud a list of words containing particular letter clusters with irregular pronunciations (e.g. the *ou* pronunciation in *touch*). This was based on a similar list used by other researchers working in the field of dyslexia [34] (results in Table 5).

### Experimental design and procedure

#### Experiment 1

A horizontal array of dot strings was presented on each trial (Figure 2); the stimuli were designed to require horizontal saccades from dot group to dot group, but did not contain any linguistic content and omitted fine-grained features comparable to the constituent parts of letters (e.g., ascenders and descenders). Participants were instructed to fixate a cross, presented on the left side of the screen for one second. The fixation cross was then replaced by a row of dot string targets and participants were required to scan, from left to right fixating each of the strings in turn, treating each dot string, in turn, as a target for the next saccade. The resulting eye movement behaviour was similar to that which occurred during reading (see Figure 3; also see Table 1). Each dot extended 0.29° of a visual angle, and was presented in white on a black background. The display remained on the screen for an experimentally determined period that allowed ample time for each of the strings to be fixated at least once (5000 ms for single dots; 10000 ms for two dot strings; 8000 ms for four dot strings; 5000 ms for six dot strings).

#### Experiment 2

Sentences were presented in white on a black background in Courier New font size 14. They were constructed with simple syntactic structures, so that both groups of children could read and understand them. The sentences were carefully screened on a group of 7 to 8 year old typically developing children who did not take part in the eye tracking experiment (all were able to read the sentences easily, indicating that the stimuli were appropriate). Practise sentences were included prior to the experiment and comprehension questions were randomly distributed throughout the experiment to check children's comprehension. Participants were instructed to read the experimental sentences for comprehension and to accurately answer occasional questions about the sentence they had just read. Scores on comprehension questions demonstrated that all participants were able to understand the sentences (adults 98% correct, typically developing children 94% correct, and children with dyslexia 88% correct; *F* (2, 24) = 2.41, *p* = .11).

#### Apparatus

Two Dual Purkinje Image eye trackers were used to record binocular eye movements. A computer was interfaced with the eye trackers, and all stimuli were presented on a 20″ monitor at a viewing distance of 100 cm. Calibrations were performed monocularly (e.g. when calibrating the left eye, the right was occluded and vice versa; for a discussion on the importance of monocular calibrations for binocular research, see 10) and following every three trials the calibration accuracy was checked monocularly for each eye and the trackers re-calibrated where necessary.
